# Mechanochemical Coupling in the Myosin Motor Domain. I. Insights from Equilibrium Active-Site Simulations

**DOI:** 10.1371/journal.pcbi.0030021

**Published:** 2007-02-09

**Authors:** Haibo Yu, Liang Ma, Yang Yang, Qiang Cui

**Affiliations:** 1 Department of Chemistry, University of Wisconsin, Madison, Wisconsin, United States of America; 2 Theoretical Chemistry Institute, University of Wisconsin, Madison, Wisconsin, United States of America; University of Arizona, United States of America

## Abstract

Although the major structural transitions in molecular motors are often argued to couple to the binding of Adenosine triphosphate (ATP), the recovery stroke in the conventional myosin has been shown to be dependent on the hydrolysis of ATP. To obtain a clearer mechanistic picture for such “mechanochemical coupling” in myosin, equilibrium active-site simulations with explicit solvent have been carried out to probe the behavior of the motor domain as functions of the nucleotide chemical state and conformation of the converter/relay helix. In conjunction with previous studies of ATP hydrolysis with different active-site conformations and normal mode analysis of structural flexibility, the results help establish an energetics-based framework for understanding the mechanochemical coupling. It is proposed that the activation of hydrolysis does not require the rotation of the lever arm per se, but the two processes are tightly coordinated because both strongly couple to the open/close transition of the active site. The underlying picture involves shifts in the dominant population of different structural motifs as a consequence of changes elsewhere in the motor domain. The contribution of this work and the accompanying paper [36] is to propose the actual mechanism behind these “population shifts” and residues that play important roles in the process. It is suggested that structural flexibilities at both the small and large scales inherent to the motor domain make it possible to implement tight couplings between different structural motifs while maintaining small free-energy drops for processes that occur in the detached states, which is likely a feature shared among many molecular motors. The significantly different flexibility of the active site in different X-ray structures with variable level arm orientations supports the notation that external force sensed by the lever arm may transmit into the active site and influence the chemical steps (nucleotide hydrolysis and/or binding).

## Author Summary

The hydrolysis of Adenosine triphosphate (ATP) provides the energy for most life processes, including the motility of molecular motors. How the chemical energy of hydrolysis is converted into mechanical work in these fascinating “nanomachines” is a central question that has only been answered in an outline form for almost all molecular motors. The fundamental challenge is that the working cycle of molecular motors involves processes of different physicochemical natures and scales, including ATP chemistry and protein structural transitions of diverse magnitudes (from a few Angstroms to a few nanometers), which makes mechanistic analysis using experiments alone difficult. Combined with previous computational studies from this lab, molecular dynamics simulations help identify energetic and structural properties of myosin, a prototypical molecular motor, that are essential to its energy conversion function. In addition to the role of flexibilities at the domain scale, which has been emphasized in previous studies of similar systems, the current results highlight the comparable significance of local flexibilities and how these flexibilities at different scales are modulated by ATP chemistry.

## Introduction

Coupling between chemical events and conformational transitions is crucial for the function of many biomolecules [1]. Notable examples include molecular motors [2–4], signaling proteins [5], and membrane transporters [6], in which large-scale conformational transitions are often coupled to the binding and hydrolysis of Adenosine triphospate (ATP) or phosphorylation. Despite many previous efforts from both experimental and theoretical studies [2–4,7,8], the complex nature of the phenomena still makes it an outstanding challenge to establish a concrete understanding of “mechanochemical coupling” in those “molecular machines” with structural and energetic details.

One particularly interesting question in this context is whether the ATP hydrolysis reaction itself is capable of driving any major structural transitions. Although the answer appears to be positive according to most biochemistry textbooks [1], the popular modern view in the biophysical community [2,9,10] is that the large-scale structural transitions implicated in most “molecular machines” are directly coupled to the binding of ATP, and the hydrolysis of ATP mainly functions as a switch to allow the system to move forward into the next functional cycle. Indeed, considering the highly charged nature of ATP (or ATP·Mg^2+^), it is physically reasonable to expect the binding process to involve substantial local structural adjustments that can then propagate into larger structural transitions. The hydrolysis of ATP, by contrast, involves only subtle structural changes and therefore may not be particularly capable of driving major structural transitions.

Along this line, conventional myosin (which will be referred to as myosin-II or myosin in the subsequent discussions) is interesting to study because it is a prototypical molecular motor [11] that utilizes ATP; more importantly, there is solid experimental evidence [12] that supports the explicit role of ATP hydrolysis in driving the key structural transition (see below).

**Figure 1:**
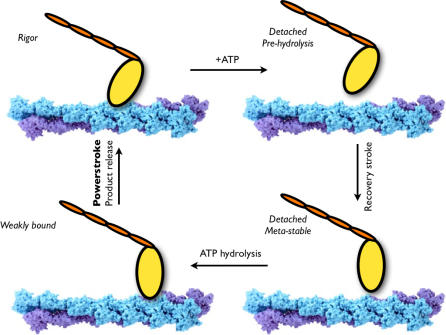
The Lymn–Taylor Functional Cycle of Myosin-II–Actin [13–17] Only a myosin monomer is shown for simplicity. The binding of ATP to the actin–myosin complex (the “rigor state”) leads to rapid dissociation of myosin from actin without immediate hydrolysis of ATP. Coupled with a major structural change in the orientation of the lever arm (recovery stroke), ATP hydrolysis proceeds, after which the motor domain rebinds to actin weakly. Following the release of P*_i_,* the motor domain undergoes “powerstroke” during which the orientation of the lever arm changes back to that in the “rigor state” and the motor domain becomes strongly bound to actin. Dissociation of ADP leads the system back to the “rigor state.” Note that the sum of free-energy drops in an entire cycle is equal to the hydrolysis free energy of ATP in *solution,* which is the ultimate thermodynamic driving force of the system.

**Figure 2:**
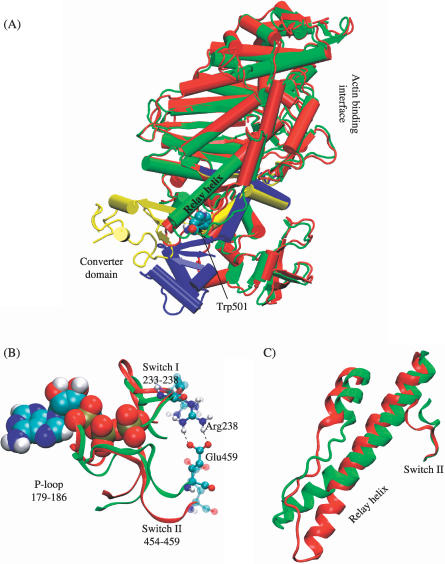
Structural Differences between Conformations of the D. discoideum Myosin Motor Domain (A) The difference between the post-rigor (1FMW [18]) and pre-powerstroke (1VOM [19]) states. The structures are aligned based on backbone atoms in the first 650 residues; red and blue correspond to 1FMW, green and yellow correspond to 1VOM. Trp501 is shown in the van der Waals form. (B) Superposition of the active-site region in 1FMW and 1VOM (same color coding as in (A); the nucleotide is shown in the van der Waals form). (C) Superposition of the relay helix and relay loop in 1FMW and 1VOM with the same color coding as in (A).

The fundamental functional cycle of myosin-II has been well-established via kinetic studies and is summarized in Figure 1 [13–17]. The ATP hydrolysis step implicates the detached kinetic states, for which relevant high-resolution X-ray structures are available for the truncated motor domain. As shown in Figure 2A, two representative X-ray structures were solved with ATP (PDB code 1FMW [18]) and ADP·VO_4_^−^ (PDB code 1VOM [19]) as the ligands, respectively, and they were postulated to reflect the conformations before and after (or during) ATP hydrolysis, correspondingly. Fitted into the kinetic scheme of the functional cycle (Figure 1), they are referred to as the “post-rigor” and “pre-powerstroke” states, respectively. The most visible structural change occurs in the C-terminal converter region, which undergoes a major rotation between the two structures and is believed to propagate into the lever-arm rotation; with the two structures best-fitted based on the first 650 residues, the backbone atom root mean square deviation (RMSD) in the converter is as large as 18.7 Å while it is only 2.4 Å for the rest. In addition to this striking rearrangement, there are also more subtle structural changes in the nucleotide binding site (Figure 2B) and the “relay helix” (Figure 2C) that connects the binding site and converter.

There has been a large body of experimental [12, 17–28] and computational [29–32] studies aimed at understanding the mechanism of the “recovery stroke” between the post-rigor and pre-powerstroke states and its connection to the hydrolysis of ATP. The FRET study of Shih et al. [12] has firmly illustrated that the rotation of the converter, which is the major part of the recovery stroke, is dependent on and tightly coupled to the hydrolysis of ATP. This is consistent with the functional cycle of myosin (Figure 1): (i) the binding of ATP is used to detach the motor domain from the actin and therefore the recovery stroke needs an alternative driving force; (ii) without a tight coupling between the hydrolysis and recovery stroke, the motor may rebind to the actin with a lever-arm configuration that is incapable of performing the powerstroke, which leads to futile ATP hydrolysis.

An interesting point further arises when one considers the energetics of the functional cycle (see Discussion). Since ATP hydrolysis occurs in the detached kinetic states, which are not capable of exerting force on actin and performing work, the reaction is expected to have a small free-energy drop [33], which is consistent with experimentally measured equilibrium constants [14,28]. Naively, the nearly thermoneutral nature of the hydrolysis reaction seems inconsistent with its observed role [12] in driving the major structural transitions in the recovery stroke.

Therefore, a question of critical functional importance is: what are the properties of the myosin motor domain that ensure a tight coupling between ATP hydrolysis and the converter rotation, despite not only the long distance separating the nucleotide binding site and the converter but also the nearly thermoneutral nature of the hydrolysis reaction? To answer this question, it is important to address not only the structural features of the active site but also the energetics of various processes involved in the recovery stroke. In this work, we meet this challenge with a combination of equilibrium simulations of the myosin motor domain. Specifically, potential of mean force (PMF) simulations [34,35] are used to quantitatively characterize the open/closed transition of the nucleotide binding site and analyze how this transition is coupled, thermodynamically, to the structure of the relay helix and orientation of the converter domain. Equilibrium simulations, using both molecular mechanics (MM) and combined quantumn mechanics/molecular mechanics (QM/MM), are also carried out for different nucleotide states (ATP versus ADP·P*_i_*) to probe the effect of ATP hydrolysis on the active-site behavior, which aims to explore how ATP hydrolysis might induce the subsequent rebinding of the motor domain to actin (Figure 1).

In the accompanying article [36], several alternative computational approaches, which include targeted molecular dynamics (MD) [37,38], normal mode [39,40] hinge analysis, and statistical coupling analysis [41,42] are used to explore transition pathways for the recovery stroke and to identify residues important to the coupling between distant functional sites.

Compared with previous simulation studies on the mechanochemical coupling in myosin [29–32,43] and other molecular motors [8,44–46], the present set of studies represents the first serious attempt to examine energetic properties concerning the conformational transitions in these complex systems and a *systematic* analysis of residues critical to mechanochemical coupling. With all the results taken together, a clearer mechanistic picture emerges for the coupling between ATP hydrolysis and various essential structural motifs, which includes some features that might be shared by different classes of motors.

## Results

To facilitate discussion, we summarize the nomenclature and conformational characteristics for key structural motifs in Table 1 following the discussions in previous structural studies [17,47]. Specifically, we refer the active site with the critical salt bridge (Arg238-Glu459) formed as the *closed* configuration; otherwise as the *open* configuration. In the 1FMW structure, the converter domain is in a *down* position with the relay helix straight, while in the 1VOM structure, the converter domain is in an *up* position with a kink in the relay helix (see Table 1 and Figure 2A).

**Table 1:**
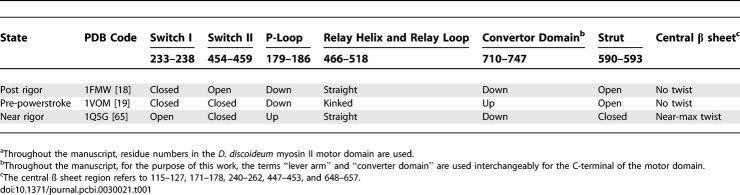
Structural Characteristics for the Three Conformations of the Myosin Motor Domain Characterized with X-ray Crystallography^a^

### Energetics for the Open/Closed Transition of the Nucleotide Binding Site in Different Conformational States

Although the converter domain and the active site were seen to adopt different conformations in the two X-ray structures (1FMW and 1VOM) [18,19], to what degree these distant regions are coupled thermodynamically is not well-established; whether the nucleotide chemical state (e.g., ATP versus ADP·P*_i_*) contributes *directly* to the coupling is also not clear. These issues are probed with PMF calculations for the open/closed transition in the active site with different X-ray structures of the motor domain, which mainly differ in the lever-arm orientations (down versus up) and relay helix conformations.

### Thermodynamics Based on PMF Calculations

**Figure 3:**
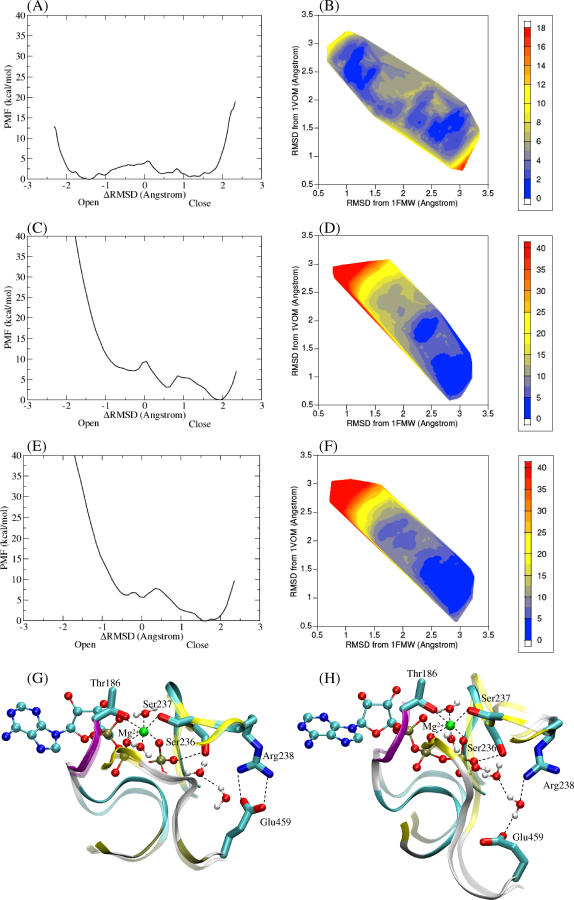
PMF Calculations for the Open/Close of the Active Site with Different Converter Orientations The reaction coordinate for 1-D PMFs is Δ*RMSD* relative to the open and closed active-site configurations; those for 2-D PMFs are *RMSD*s relative to the open and closed active-site configurations. (A,B) PMFs (in kcal/mol) for the 1FMW:ATP state. (C,D) PMFs (in kcal/mol) for the 1VOM:ATP state. (E,F) PMFs (in kcal/mol) for the 1VOM:ADP·P*_i_* state. (G) Superimposition of the final structure from the 1FMW:ATP simulation at Δ*D_min_* = 2.2 Å (opaque) and the closed reference structure (transparent). (H) Superimposition of the final structure from the 1VOM:ATP simulation at Δ*D_min_* = 2.2Å (opaque) and the open reference structure (transparent).

The PMFs for the open/closed transition of the active site have been calculated in both one dimension (*ΔRMSD*) and two dimensions (*RMSD_FMW_*, *RMSD_VOM_*); see Methods. The general trend is very similar; thus, we will only refer to the 1-D PMFs in the discussion, although both sets are shown in Figure 3 for comparison.

With the lever arm down (1FMW:ATP), the PMF has the double-well feature, with the open configuration slightly preferred (∼−0.6 kcal/mol) and a low barrier of ∼5 kcal/mol (Figure 3A and 3B). Experimentally it was found that Switch II closure is a rapid (∼1000 s^−1^) event with an equilibrium constant close to 1 (Δ*G* ^0^ ∼ 0 kcal/mol) [14,28]. The drastic difference between the measured open/closed rate (∼1000 s^−1^) and the calculated barrier (∼5 kcal/mol, which corresponds to a rate of 1.4 × 10^9^ s^−1^ using a pre-factor of *k_B_T/h* in a transition state rate expression, ignoring the effect of damping) might be due to the approximate nature of the reaction coordinate used in the PMF calculations; an alternative explanation is that the reporting Trp501 fluorescence might be more directly related to the position of the lever arm rather than the open/closed of the Switch II motif [48] (also see [36]). Nevertheless, the trend that the open and closed configurations of the active site have similar energetics in post-rigor is found in both experiments [27] and the present PMF calculations.

When the lever arm is up (1VOM:ATP, 1VOM:ADP·P*_i_*), the free-energy well corresponding to the open active site (*ΔRMSD* ∼ −1.5 Å) disappears and the closed state becomes the much preferred one (Figure 3C–3F). The PMF profile is qualitatively the same with ATP and ADP·P*_i_* bound, although the open configuration (*ΔRMSD* ∼ −1.5 Å) appears to be even higher in free energy in the latter case; this is in qualitative agreement with the expectation based on QM/MM studies of ATP hydrolysis in different active-site conformations that ADP·P*_i_* favors the closed configuration compared with ATP [29,49]. Due to the high energy cost, the open configuration of the active site is essentially inaccessible when the lever arm is up, regardless of the nucleotide chemical state. In other words, the lever-arm orientation is clearly tightly coupled to the active-site conformation, presumably through residues in the relay helix (see below).

### Behavior of Individual Residues

It is worth noting that the free-energy minimum corresponding to the closed active site has an *RMSD* ∼ 1.5 Å (Figure 3G and 3H) relative to the 1VOM X-ray structure in all three sets of simulations shown in Figure 3, which suggests that the Δ*RMSD* reaction coordinate offers an unbiased description for the open/closed transition in different X-ray structures. The effectiveness of the simulation protocol is also demonstrated by the water structure in the active site. As emphasized in previous simulations and experimental studies [22,29], there are two water molecules in the closed active site and they form stable hydrogen bonds with a number of residues and the γ-phosphate of ATP; this hydrogen-bonding network, which does not exist in the open configuration of the active site, helps to orient the lytic water for a favorable nucleophilic attack. Encouragingly, the correct hydrogen-bonding network is formed spontaneously in 1FMW:ATP simulations when the active site is closed with umbrella sampling simulations based on Δ*RMSD* (Figure 3G).

**Figure 4:**
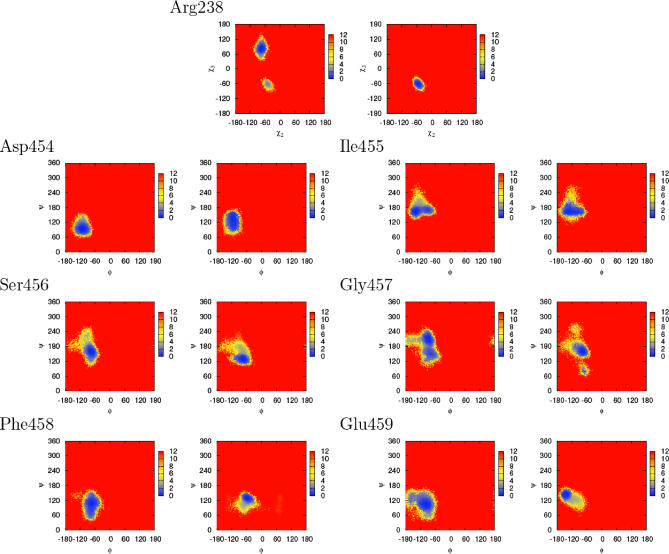
Free-Energy Map (in kcal/mol) for the Conformations of Arg238 Sidechain (χ_2_, χ_3_) and Main Chains (ϕ, ψ) in the Switch II Region (454–459), Calculated Using Equations 5 and 6 For each residue, map on the left is for the 1FMW:ATP state and map on the right is for the 1VOM:ATP state.

Although the active site and the converter are separated by more than 40 Å, the different active-site flexibilities reflected by the open/closed PMFs indicate that variation in the lever-arm orientation produces changes that propagate to the active site, which in turn affects the relative stability of the open and closed configurations of the active site. To identify residues that make major contributions to the open/closed PMF and their dependence on the lever-arm orientation, free-energy maps for local torsions of active-site residues are obtained using Equations 5 and 6. The (χ_2_, χ_3_) conformational free-energy profile for Arg238 and (ϕ, ψ) conformational free-energy profiles for residues Asp454, Ile455, Ser456, Gly457, Phe458, and Glu459 are shown in Figure 4. As references, the dihedral angles describing the conformation of those residues in the crystal structures are summarized in Table 2.

**Table 2:**
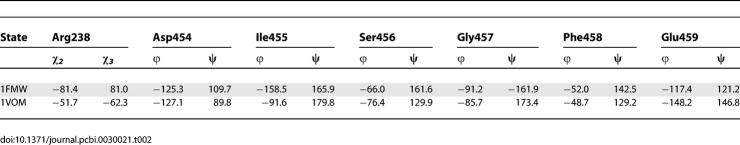
Dihedral Angles (in Degrees) for Arg238 and Switch II Residues in X-Ray Structures 1FMW [18] and 1VOM [19]

The main chain conformational spaces accessed in the PMF simulations are within allowed regions in the Ramachandran plot [50,51], which reassures us that the sampled conformations do not involve unphysical ones. The most striking difference between the 1FMW:ATP and 1VOM:ATP simulations is the Ramachandran plot for the side-chain conformation of Arg238, which involves flipping around χ_3_ during the open/closed transition. When the lever arm is down, the side chain can adopt two different conformations about (−80°, 90°) *(A)* and (−50°, −60°) *(B)*. Region *A* is preferred over region *B* with a free-energy difference of ∼3 kcal/mol. When the lever arm is up (1VOM), by contrast, region *A* is no longer accessible. All other residues within Switch II involve a population shift between different conformations, most notably Gly457 and Glu459. This is consistent with the proposal that Gly457 is involved in a rotation to allow the formation of the Arg238-Glu459 salt bridge and to make an amide hydrogen bond to the γ-P of ATP during the Switch II closure [15]; a mutation of Gly457 to even an Ala affects the flexibility in this region and therefore reduces the stabilization of the γ-P, which in turn causes the loss of the basal ATPase activity [52].

**Figure 5:**
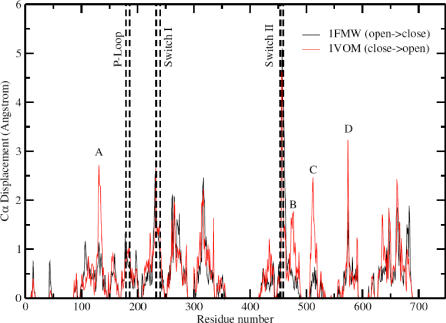
Displacements of the Cα Atoms in the PMF Simulations of the 1FMW:ATP and 1VOM:ATP States, Relative to the Corresponding X-ray Structure The structures are taken as the last snapshot from PMF simulations at Δ*D_min_* = 2.2 Å, or at Δ*D_min_* = −2.2 Å. Region A, the C-terminal of the second central ß sheet; Region B, the loop between the N-terminal of the relay helix and Switch II; Regions C and D, the actin-binding clefts.

**Figure 6:**
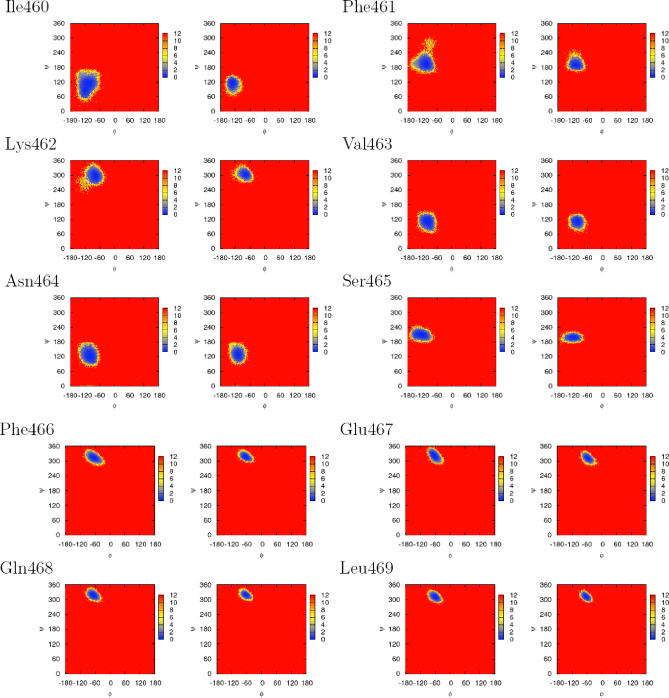
Same as Figure 4, but for Residues within the Loop between Switch II and the N-Terminal of the Relay Helix For each residue, the map on the left is for the 1FMW:ATP state and the map on the right is for the 1VOM:ATP state.

Although only Switch I/II residues are subject to bias in the umbrella sampling simulations, substantial changes in other regions of the motor domain are observed. As shown in Figure 5, opening the active site in the 1VOM:ATP simulations induces significant changes in the actin-binding interface (regions C,D), the loop between Switch II, and the N-terminal of the relay helix (region B), as well as the C-terminal of the second central ß-sheet (region A); the displacements in those regions are substantially larger than their equilibrium fluctuations (unpublished data). As illustrated by the Ramachandran (ϕ, ψ) conformational free-energy profiles (Figure 6) for the residues between Switch II and the N-terminal of relay helix (region B as defined in Figure 5), the accessible conformational space is more restricted in the lever-up (1VOM:ATP) simulations compared with the lever-down case (1FMW:ATP), which partly explains why the closed state is preferentially stabilized when the lever arm is up. For example, the (ϕ, ψ) of Ile460 in the X-ray structures of 1FMW and 1VOM are (−130°, 85°) and (−133°, 127°), respectively. With the lever arm down, Ile460 can have a widely accessible conformation space surrounding its conformations in both X-ray structures; with the lever arm up, Ile460 preferentially adopts its conformation in the closed state.

### Structural Flexibility of the Closed Active Site Before and After ATP Hydrolysis

**Figure 7:**
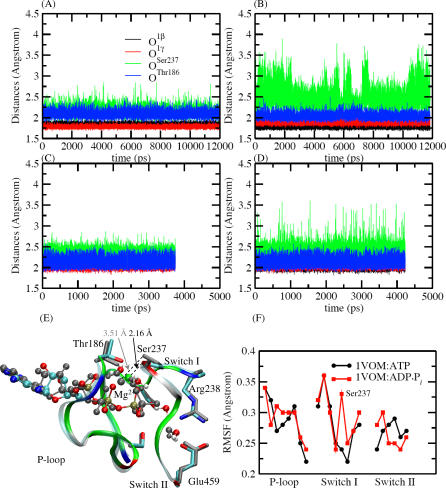
Structural Properties of the Closed Myosin Active Site During Equilibrium MD Simulations with Different Nucleotide Chemical States (ATP or ADP·P*_i_*) (A–D) Instantaneous distances between Mg^2+^ and oxygen atoms in its four nonwater ligands. (A) MM ATP state. (B) MM ADP·P*_i_* state. (C) QM/MM ATP state. (D) QM/MM ADP·P*_i_* state. O^1ß^ refers to O^1ß^ in ATP or ADP; O^1γ^ refers to O^1γ^ in ATP or the closest oxygen atom in P*_i_*; O^Ser237^ refers to O^γ^ in Ser237; O^Thr186^ refers to O^γ2^ in Thr186. (E) Overlay of the active site in two snapshots from MM simulations with ATP and ADP·P*_i_*, respectively. The one with color coding is for the ATP state while the one in grey is for the ADP·P*_i_* state. The Mg^2+^-O^Ser237^ distance is much longer in the ADP·P*_i_*-state snapshot, due to displacements in both the Ser237 sidechain and the hydrolyzed nucleotide. (F) Root mean square fluctuation (RMSF) for Cα atoms in the three critical active-site motifs based on equilibrium MM simulations. Ser237 has notably larger fluctuations in the ADP·P*_i_* state, although the rest residues do not show distinct differences.

To further explore how ATP hydrolysis initiates structural changes that ultimately induce the rebinding of the motor domain to actin, substantially longer equilibrium simulations (up to 12 ns) of the closed active site are performed for the ATP and ADP·P*_i_* chemical states with both MM and QM/MM potentials. The results of MM and QM/MM simulations are largely consistent; thus, only MM values are discussed below, although both sets of results are shown in Figure 7 for comparison. We focus on the interaction between Mg^2+^-nucleotide and the Switch I region because it has been speculated that changes in Switch I are coupled to transitions in actin-binding motifs [17].

In the ATP state (Figure 7A), the two distances between Mg^2+^ and the nucleotide oxygen atoms are significantly shorter than those between Mg^2+^ and Thr186/Ser237. The average distances for O^1ß^-Mg^2+^ and O^1γ^-Mg^2+^ are 1.83 ± 0.04 Å and 1.78 ± 0.03 Å, respectively; the corresponding values for O^Ser237^-Mg^2+^ and O^Thr186^-Mg^2+^ are 2.14 ± 0.11 Å and 2.09 ± 0.08 Å, respectively. This trend is expected considering that the oxygen atoms in the nucleotide are more negatively charged (∼−0.85e) than those in polar amino acid side chains (∼−0.66e) [53].

In the ADP·P*_i_* simulations (Figure 7B), the most striking change is observed for the O^Ser237^-Mg^2+^ distance, for which the average is 2.48 Å, 0.34 Å longer than that in the ATP simulation. The fluctuation in the distance also increases more than twice that in the ATP state, to 0.25 Å. The largest distance sampled in ∼12 ns reaches almost 4 Å, as compared with 2.75 Å in the ATP simulations. In the QM/MM simulations (Figure 7C and 7D), the Mg^2+^-O distances are consistently shorter, but the trend of a weaker interaction between Mg^2+^ and Ser237 in the ADP·P*_i_* simulation is also evident. Therefore, both MM and QM/MM simulations show that following ATP hydrolysis the interaction between the Mg^2+^-nucleotide and Switch I becomes substantially weakened; the fact that MM simulations produce similar results as QM/MM simulations suggests that the cause is largely electrostatic in nature and due to the difference in the charge distribution in the nucleotide before and after hydrolysis. A careful examination of the structures in the simulations suggests that the increase in the distance has contributions from both the structural relaxation of Mg^2+^-nucleotide following hydrolysis and the slight displacement of the Ser237 side chain (Figure 7E); the average backbone configuration of the Switch I motif, however, is not perturbed significantly during the nanosecond scale simulations (unpublished data). Nevertheless, the weakened interaction between Mg^2+^-nucleotide and Ser237, as reflected by the larger fluctuations of Ser237 (Figure 7F), is expected to make structural transition of Switch I more facile. Analysis of sidechain internal fluctuations also indicates that Ser237 is the only residue that is affected significantly by hydrolysis (see Protocol S1).

## Discussion

### Defining Mechanistic Questions Regarding Mechanochemical Coupling Based on Functional Constraints

Before discussing the results from the current work in detail, it is useful to recapitulate the fundamental questions regarding mechanochemical coupling in myosin, and other molecular motors, that ultimately drive our study. As pointed out elegantly by Hill and others in their pioneering analysis of coupled biological processes [33,54,55], biological systems need to meet specific thermodynamic and kinetic constraints to acquire the corresponding characteristics necessitated by their biological functions. Specifically for myosin, the constraints based on functional considerations are [55]: (i) processes that occur in the detached states have small free-energy drops; (ii) in particular, the ATP hydrolysis reaction itself is close to being thermoneutral; (iii) the ATP hydrolysis step is tightly coupled to the reorientation of the lever arm. The first two constraints arise because for a motor to achieve a high thermodynamic efficiency, only the steps capable of performing useful work should be associated with large free-energy drops, since no work can be done in the detached states (no force can be exerted on the actin), and, considering that the sum of free-energy changes through a functional cycle is strictly a constant (the hydrolysis free energy of ATP in solution), the detached states should have small free-energy drops. In principle, large free-energy increases and drops associated with the detached states may cancel out to still leave a large free-energy drop for the powerstroke, but the large free-energy increases for certain step(s) may compromise the speed of the motor; in other words, meeting the first two constraints is likely correlated with a fast motor without any significant kinetic bottleneck. In this context, we emphasize that one has to distinguish the free-energy drop for ATP hydrolysis in the motor domain, which is small in magnitude (as shown experimentally for not only myosin [14] but also a number of other motors such as F_1_-ATPase [56]), from that for the entire functional cycle; the latter is strictly equivalent to the hydrolysis free energy of ATP in solution and forms the ultimate thermodynamic driving force for the vectorial motion of the motor. The third constraint minimizes the amount of futile ATP hydrolysis, because otherwise a loose coupling may lead to rebinding of the motor to actin with a lever-arm orientation incapable of powerstroke.

Although these “idealized” constraints are derived by considering a motor with the optimal efficiency, and most biological motors do not need to have perfect thermodynamic efficiency for their biological functions [7], they provide an interesting set of guidelines to investigate the properties of molecular motors in the context of mechanochemical coupling. Indeed, it is of interest to investigate to what degree these constraints are satisfied in realistic systems and, if so, how they are implemented in structural and energetic terms [49,57].

### Constraints I and II in the Myosin Motor Domain

For constraint I, although our study here explicitly deals with only a few steps in the functional cycle, a number of relevant observations can be made. First, normal mode analyses of multiple conformational states clearly demonstrate that the structural transitions are highly correlated with the low-frequency modes and, therefore, intrinsic structural flexibility of the motor domain [36,43]. Although no explicit free-energy cost can be inferred from such normal mode analyses, the significant degree of observed correlation strongly suggests that conformational transition among those states is associated with a modest free-energy change. A series of hydrophobic and polar interactions is observed in both minimum energy path [30] and biased MD simulations [36] to facilitate the large structural rearrangements in the relay helix and converter domain and ensures the corresponding low energy cost. Second, for the open/close of the active site, which has been shown to activate ATP hydrolysis [22,29,49], both current PMF calculations and recent fluorescence experiments [27] suggest that the transition is nearly thermoneutral with the lever arm down (the expected orientation following detachment from actin). The PMF calculations are worthwhile because the interpretation of the fluorescence is not straightforward and the signal due to Trp501 might be better correlated with the lever-arm position rather than the active-site configuration per se [48]. Third, for processes other than the powerstroke, although not explicitly studied here, it is natural to envision how they can be nearly thermoneutral. The detachment of the motor domain from actin is compensated for by the binding of ATP; the rebinding of the motor domain to actin following ATP hydrolysis is known to be only a weak association; the weak → strong actin-binding transition is compensated for with dissociation of hydrolysis product(s) and may partially couple to the powerstroke.

For constraint II, which is consistent with the measured equilibrium constant [14,27], both our [29] and others' [31] QM/MM simulations suggest that the small free-energy drop is likely controlled by well-orchestrated electrostatic environment of the active site. Both favorable and unfavorable contributions from residues/water in the closed active site have been identified, which together result in a small hydrolysis-free energy. Importantly, as argued below, the small free-energy drop for the hydrolysis step itself does *not* compromise the coupling between ATP hydrolysis and lever-arm orientation as far as both couple tightly to the open/close transition of the active site.

### Constraint III in the Myosin Motor Domain

As the major focus of this work, understanding how constraint III is implemented is fascinating because of its allosteric nature: ATP hydrolysis in the active site is strongly coupled to the rotation of the converter domain (thus, lever arm) at 40 Å away. It is physically reasonable to argue that the two processes are coupled *indirectly* by both being *directly* coupled to variations in the active-site configuration. Indeed, both structural studies [19,22] and QM/MM simulations [29] support the idea that ATP hydrolysis is only possible with the closed active site and is prohibited in the open active site. In addition, PMF calculations (Figure 3) show explicitly that the active site open/close transition is sensitive to the conformation of the lever arm and relay helix. In the post-rigor state (lever arm down, relay helix straight), the open/close transition is a rapid and thermoneutral process, while with the pre-powerstroke state (lever arm up, relay helix kinked) the active site is strongly biased (by more than 15 kcal/mol) toward the closed configuration.

**Figure 8:**
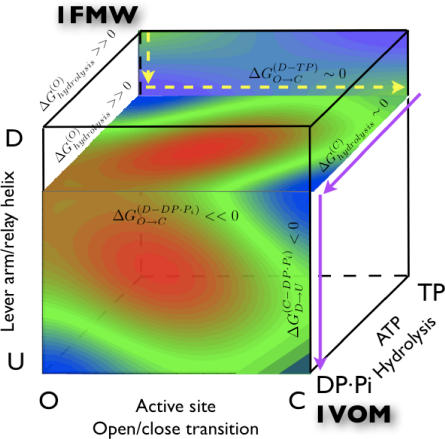
A Schematic Illustration of Thermodynamic Coupling between Processes Involved in the Myosin Detached States (Characterized by the PDB codes 1FMW, 1VOM; see Table 1) The open(O)/close(C) transition of the active site, the hydrolysis of ATP (TP versus DP·P*_i_*), and the rotation of the converter domain (Down versus Up); different converter orientation is presumed to be correlated with different relay helix conformations as implied in the X-ray structures. The schematic free-energy maps (red indicates high-energy region, blue low-energy region, and green intermediate-energy region) are sketched based on results from the previous QM/MM simulations [29], current PMF simulations, and thermodynamic cycle considerations. The dashed and solid arrows indicate a likely sequence of events during the 1FMW → 1VOM transition.

Taken together, at the thermodynamic level, the simulation results show explicitly how ATP hydrolysis is coupled to the lever-arm rotation through a series of population shifts in local conformations (Figure 8). Starting with the post-rigor conformation, the active site (more precisely, Switch II) rapidly explores the open and closed configurations; with the closed configuration, ATP hydrolysis is a relatively rapid and reversible process. Based on the thermodynamic linkage between the hydrolysis and active-site closure (see the O-C-DP·P*_i_*-TP plane in Figure 8), the dominant population of the active site is shifted toward the closed configuration once ATP is hydrolyzed (with ADP·P*_i_* bound). In turn, based on the thermodynamic linkage between the active-site open/close transition and the converter rotation (see the D-U-O-C plane in Figure 8, assuming that the converter and relay helix are tightly coupled), closure of the active site drives the dominant population of the lever arm from the post-rigor to the pre-powerstroke orientation.

Although this description is purely thermodynamic in nature and does not specify the sequence of events (see discussions in the accompanying paper [36]), the clear energetic relations make it possible to refine models concerning the functional cycle, which is difficult to do with a structure-only analysis [30]. For example, most kinetic schemes in the literature assume that activating the hydrolysis depends on (at least partial) rotation of the lever arm [12], because it is difficult in experiments to simultaneously monitor the active-site motion and lever-arm rotation due to their significantly different length scales [26,27,48]. Our computational analyses taken together, however, suggest that the activation of ATP hydrolysis does not require the rotation of the lever arm per se, because closure of the active site, which directly regulates ATP hydrolysis, is a facile process even with the lever arm in the post-rigor orientation. This is consistent with the phenotype of many “uncoupling mutants” [23,24], in which the ATPase activity is similar to the wild-type but motility (which presumably is correlated with lever-arm rotation) is significantly compromised or abolished.

As mentioned in the Introduction, the tight coupling between ATP hydrolysis and converter rotation is even more striking considering the near-thermoneutral nature of processes in the detached states. With the energetics-based analysis, it is worth noting that a nearly thermoneutral ATP hydrolysis (in the motor) does *not* imply a weak coupling to lever-arm orientation. Provided that the lever arm is strongly correlated to the active-site open/close transition through the relay helix, the only requirement for ATP hydrolysis (for the sake of avoiding futile hydrolysis) is that it is tightly coupled to the same active-site transition; the magnitude of this coupling depends only on the *relative* hydrolysis free energy in the open and closed active-site configurations. As a near-thermoneutral reaction in the closed state but unfavorable reaction in the open state, the ATP hydrolysis is strongly coupled to the open/close transition and, simultaneously, satisfies the thermodynamic constraint II imposed on the motor.

Another point clearly underlined by the energetics-based analysis is that the coupling between ATP hydrolysis and the lever-arm rotation relies on the intrinsic flexibility of the motor domain at both the small (active-site) and large (converter-rotation) scales [29]. In this sense, the current description is reminiscent of the “rectified Brownian diffusion” framework [58] proposed for a number of motors including myosin. The current analysis, however, is built on an atomistic description of the relevant processes with concrete structural and energetic features; therefore, we are able to provide mechanistic details regarding how “Brownian motions” of the motor domain are “rectified” by the hydrolysis of ATP, or, in the language of physical chemistry, how the dominant population of different structural motifs is shifted by events that occur elsewhere in the motor domain. Moreover, we note that although many previous analyses of “molecular machines” based on normal modes [29,44,59–63] emphasized the role of intrinsic structural flexibility at the domain scale, the current study highlights the comparable significance of local flexibility (e.g., active site) and, more importantly, how these flexibilities at different scales are *modulated* by chemistry. Conversely, the significantly different flexibility of the active site, as captured by the PMF calculations, in different X-ray structures of the motor domain, supports the notion that external force sensed by the lever arm may transmit into the active site and perturb processes such as ATP hydrolysis or product release, which ultimately leads to the change in the motor speed [64].

### Communication between Active Site and the Actin-Binding Interface

In addition to the coupling between hydrolysis and the converter, an issue of major importance concerns the coupling between the active-site activities and the actin interface. In the Lymn−Taylor scheme [13–15], hydrolysis of ATP leads to the reattachment of the motor domain to actin, although the corresponding mechanism is not well-understood, due largely to the lack of a high-resolution structure for the myosin−actin complex. The current set of equilibrium simulations of the active site, although limited by the nanosecond time scale and the boundary condition, have provided useful hints.

First, significant distortion of the actin-binding interface and the central ß sheet has been observed to be induced by the opening of the active site in the umbrella sampling simulations (Figure 5), strongly suggesting the coupling between the open/close of the active site and motion in the actin-binding interface. This hypothesis is consistent with the structural analysis of Geeves and Holmes [17], who proposed that during the powerstroke the opening of Switch I induces the twisting of the central ß sheet as well as the closure of the actin-binding cleft.

In addition, an interesting observation from simulations with different nucleotide chemical states found that the interaction between the Mg^2+^ and Switch I (in particular, Ser 237) is substantially reduced once ATP is hydrolyzed; this is observed with both MM and QM/MM potential functions, which suggests that the cause is largely electrostatic and structural in nature rather than due to any subtle quantum mechanical effects. This supports the hypothesis of Reubold et al. [65] and Coureux et al. [66], who analyzed the X-ray structures of nucleotide-free myosin II and myosin V and proposed that hydrolysis of ATP leads to the destabilization of the closed configuration of Switch I. This might prepare for the opening of the Switch I loop, which has been postulated to occur upon binding of actin and is necessary for the release of the hydrolysis products [17]. We note that as this work was in progress, a similar observation was reported in a QM/MM minimization study of myosin [31] and an MD simulation using MM potential [32]. Our unique contribution here is the confirmation of this important effect with both MM and QM/MM potential functions with extensive sampling. An interesting observation in [32] is that Asn475 breaks its hydrogen bond with Switch II upon ATP hydrolysis. This, however, was not observed in either the MM or QM/MM simulations here; to what extent this is due to the spherical boundary condition of these simulations is being investigated (also see Protocol S1). Interestingly, we note that Koppole et al*.* [32] used a mixture of the force-field parameters in their model, which involves polar-hydrogen parameters for nonaromatic residues and all-atom parameters for the aromatic residues. It is not clear what justifies such mixing of force-field parameters and what is the impact of this on the simulation result.

### Conclusions

Vectorial biological systems such as molecular motors and pumps are fascinating to study [57] because their unique functional characteristics may impose specific thermodynamic and kinetic constraints [33,54,55] that distinguish them from regular enzymes. With remarkable progress in structural and dynamical characterizations of complex biomolecules in both experimental and theoretical arenas, the challenge is to *establish to what degree these “idealized” constraints are satisfied and to reveal the physical and chemical principles behind their implementation and regulation in structural and energetic terms.*

Specifically for myosin II, a characteristic molecular motor, we argue that the key constraints the motor domain *is likely* to satisfy include small free-energy drops for all steps in the detached kinetic states, and that events at the active site are tightly coupled to distant structural motifs, which are consistent with a broad range of experimental observations. Molecular simulations are then carried out to reveal how these constraints, or “mechanochemical coupling,” are reflected in the structural, energetic, and dynamical features of the motor domain. Specifically in this work, equilibrium active-site simulations with explicit solvent molecules have been carried out to probe the behavior of the motor domain as functions of the nucleotide chemical state and conformation of the converter/relay helix. In conjunction with our previous QM/MM studies of ATP hydrolysis in different active-site conformations [29] and normal mode analysis of the motor domain [43], the results here help establish an energetics-based framework for understanding the mechanochemical coupling, in which the active-site closure tightly regulates both the hydrolysis activity and orientation of the converter (thus, lever arm). The flexibilities at both the small (active-site open/close) and large (converter-rotation) scales inherent to the motor domain make it possible to implement these couplings while maintaining small free-energy changes for all key changes occurring in the detached states; in this sense, the current framework is reminiscent of the “rectified Brownian motion” model of molecule motors [58]. The contribution of this work and the accompanying paper [36] is to propose the actual mechanism for “rectifying” these “Brownian motions” and residues that play important roles in the process. In addition, the significant coupling between the converter orientation/relay helix and the active-site configuration as identified by the PMF calculations provides a concrete energetics basis for how active-site activities can be affected by the external force sensed by the lever arm. It is anticipated that many motors, not only those in the myosin family, share many similar features in their mechanochemical coupling cycles.

In the near future, the results reported here together with additional simulations of the lever-arm transition will facilitate the development of an effective phenomenological model [67,68] that can simulate the entire functional cycle of myosin(s), which ultimately provide the missing link between microscopic structural and energetic details with the behavior of motors at the macroscopic length and time scales. Before such a model can be developed, however, key questions regarding myosin–actin interactions need to be answered [17,69], which requires contributions from both experimental and theoretical efforts.

## Materials and Methods

### PMF simulations with explicit solvent.

To estimate the PMF for the active-site open/close transition, umbrella sampling calculations with a stochastic boundary condition and explicit solvent molecules are carried out using the CHARMM program (c32a2 version) [70]. Three systems are simulated: post-rigor with ATP bound (denoted as 1FMW:ATP), pre-powerstroke with ATP bound (denoted as 1VOM:ATP), and pre-powerstroke with ADP and P*_i_* bound (denoted as 1VOM:ADP·P*_i_*). As starting coordinates, the X-ray structures solved by Rayment and co-workers for the Dictyostelium discoideum myosin II motor domain are used; the lever arm is down and the active site is open in 1FMW [18] (resolution 2.15 Å), while the lever arm is up and the active site is closed in 1VOM [19] (resolution 1.90 Å). The missing residues (1, 203–208, 500–508, 622–626 in 1FMW, and 1, 205–208, 711, 716–719, 724–730 in 1VOM) are modeled by using the program ModLoop [71,72]. The protein atoms and the Mg^2+^ ion in the active site are described with the all-atom CHARMM force field for proteins [53], and the water molecules are described with the TIP3P model [73]. The hydrogen atoms are added with HBUILD [74]. In all cases the system is partitioned into a 32-Å inner region centered at the geometric center of ATP, P-loop, Switch I, and Switch II (about 12,800 atoms), with the remaining portion of the system in the outer region. Newtonian equations-of-motion are solved for the MD region (within 28 Å), and Langevin equations-of-motion are solved for the buffer region (28–32 Å) with a temperature bath of 300 K [75]. All the atoms in the inner region are subject to a weak GEO type of restraining potential to keep them inside the inner sphere with the MMFP module of CHARMM; the effect of the restraint on most inner-region atoms is negligible. All protein atoms in the buffer region are harmonically restrained with force constants determined directly from the B-factors in the PDB file [75]. Langevin atoms are updated heuristically during the simulation to consistently treat protein groups and water molecules that may switch regions during the simulation. All bonds involving hydrogen are constrained using the SHAKE algorithm [76], with a relative geometric tolerance of 10^−10^, and the time step is set to 1 fs. Nonbonded interactions within the inner sphere are treated with an extended electrostatics model, in which groups beyond 12 Å interact as multipoles [77]. The entire system is heated gradually to 300 K and equilibrated for 160 ps prior to the production simulations.

To account for the electrostatics between the inner and outer region atoms and the effect of solvation, the generalized solvent boundary potential approach developed by Roux and co-workers is used [78,79]. In the generalized solvent boundary potential, the contribution from distant protein charges (screened by the bulk solvent) in the out region 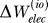 is represented in terms of the corresponding electrostatic potential in the inner region 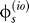 ,

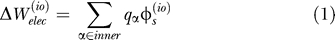
where *q_α_* is the partial charge on atom α. The dielectric effect on the interactions among inner-region atoms is represented through a reaction field term

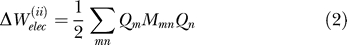
where *Q_m/n_* is the generalized multipole moment and *M_mn_* is the element of the reaction field matrix, *M*. The static field due to the outer region 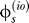 is evaluated with the linear Poisson–Boltzmann equation using a focusing scheme that place a 70-Å cube of fine grid (0.4 Å) into a larger 120-Å cube of coarse grid (1.2 Å). The reaction field matrix, *M,* is evaluated using 400 spherical harmonics. In the Poisson–Boltzmann calculations, the protein dielectric constant ɛ*_p_* = 1, the water dielectric constant ɛ*_w_* = 80, and 0.0 M salt concentration is used. The optimized radii of Roux and Nina [80,81], based on experimental solvation energies of small molecules as well as the calculated interaction energy with explicit waters, are adopted to define the solvent–solute dielectric boundary.

In the umbrella sampling simulations, intermediate structures along the transition pathway between the open and closed active site are generated by using a 1-D RMSD restraint on all atoms in the active site (Switch I: 233–238 and Switch II: 454–459). Two harmonic restraining potentials are applied to *rmsd* (**X_t_** − **X_open_**) and *rmsd* (**X_t_** − **X_closed_**), respectively, with the condition *rmsd* (**X_t_** − **X_open_**) + *rmsd* (**X_t_** − **X_closed_**) = 2.4 Å; **X_t_** is the instantaneous structure, and **X_open_** and **X_closed_** are the two reference configurations of the active site based on the 1FMW and 1VOM X-ray structures. The intermediates are generated at 0.1-Å RMSD intervals, which produced 25 intermediate structures spanning the range of ∼2.4 Å RMSD separating the two reference structures. These intermediate structures are then used as the starting points for umbrella sampling simulations [34,35,82] with the restraining potential *w_j_* on *ΔRMSD* as a reaction coordinate [83,84]

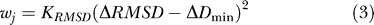
where Δ*D_min_* specifies the position of the minimum for the harmonic umbrella potential in a specific window. The Δ*RMSD* is the difference between the all-atom RMSD values for the Switch I and Switch II regions of the instantaneous structure (**X_t_**) from the two reference structures (**X_open_** and **X_closed_**),

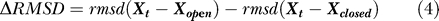
The force constant *K_RMSD_* is gradually reduced from 500 kcal/(mol·Å^2^) to 25 kcal/(mol·Å^2^) in the 50-ps equilibration simulation for each window and kept fixed for another 100 ps of production simulation. Additional windows are added or additional simulations are performed for some windows by inspecting the 2-D distribution of RMSD, *ρ_bias_* (*rmsd* (***X*_*t*_** − ***X*_*open*_**), *rmsd* (***X*_*t*_** − ***X*_closed_**)). The Weighted Histogram Analysis Method (WHAM) [82,85,86] is used to obtain the PMF along the 1-D reaction coordinate (Δ*RMSD*) from the instantaneous values of *ΔRMSD* saved every step during the MD simulations. The convergence of the PMF profiles is checked by calculations using substantially longer trajectories, up to 450 ps per window and in total about 11 ns (see Protocol S1).

The MD simulations with the umbrella potential applied along Δ*RMSD* as the reaction coordinate contain information about many individual degrees of freedom that contribute to the overall transition between the open and closed states. It is possible to estimate the free-energy profile along other additional degrees of freedom using trajectories from these umbrella sampling MD simulations [83,84]. For example, one can obtain the PMF surface as a function of the 2-D reaction coordinates defined by (*rmsd* (***X*_*t*_** − ***X*_*open*_**) and (***X*_*t*_** − ***X*_closed_**). The procedure to obtain such PMF begins by considering the biased probability distribution *ρ_bias_*(*ξ*_1_, *ξ*_2_, *ξ*_3_) where *ξ*_1_ = *rmsd* (***X*_*t*_** − ***X*_*open*_**), *ξ*_2_ = *rmsd* (***X*_*t*_** − ***X*_closed_**), and *ξ*_3_ = Δ*RMSD*. The unbiased probability distribution *ρ*(*ξ*_1_, *ξ*_2_, *ξ*_3_) can be obtained using the standard Weighted Histogram Analysis Method approach (by setting the biased potential in *ξ*_1_, *ξ*_2_, direction to zero). The probability *ρ*(*ξ*_1_, *ξ*_2_) can then be obtained by integrating *ρ*(*ξ*_1_, *ξ*_2_, *ξ*_3_) along *ξ*_3_:

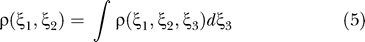
and the corresponding PMF surface can be then obtained from

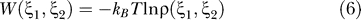



In a similar way, the PMF along the local (ϕ, ψ) space for some key residues are calculated from the trajectories saved every 100 steps during the MD simulations.

### Equilibrium MD simulations of the closed state with different nucleotides using MM and QM/MM potentials.

To probe the effect of ATP hydrolysis on the active-site structure, especially the Switch I region that has been proposed to communicate with the distant actin-binding interface, we carry out both MM and QM/MM [87,88] MD simulations for the pre-powerstroke state (1VOM [19]) with different nucleotides (ATP or ADP·P*_i_*). QM/MM simulations are also carried out to investigate if the qualitative behavior is sensitive to the treatment of charge transfer and polarization associated with the nucleotide.

The simulation setup is essentially the same as that in the PMF simulations described above, except that a smaller inner region of 20-Å radius is used to allow much longer simulations of the active site (∼12 ns for MM and ∼4 ns for QM/MM simulations). In the QM/MM simulations, 48 atoms are described quantum mechanically, including tri-phosphate and part of the ribose group of ATP, the lytic water (or the corresponding atoms in ADP·P*_i_*), the Mg^2+^ ion and all its ligands (the side chains of Thr186, Ser237, and two water molecules), as well as the side chain of the conserved Ser236. These atoms are described at the SCC-DFTB level [89,90] with the recent parameterization for phosphate systems (Yang et al., unpublished data). Four link atoms are introduced to saturate the valence of the QM atoms at the QM/MM interface [88], which interact with all MM atoms electrostatically except with the link host (Cα atoms in Thr186, Ser236, Ser237, and C5′ in the ribose); no van der Waals interactions are considered for the link atoms. This link atom treatment has been shown to work well when the nearby MM charges are small [91–93], which is the case here. The generalized solvent boundary potential approach is used for treating electrostatics in both MM [78,79] and QM/MM [94] simulations.

## Supporting Information

Protocol S1 Appendix (405 KB PDF)

## Abbreviations

ATP: Adenosine triphosphate
MD: molecular dynamics
MM: molecular mechanics
PMF: potential of mean force
QM/MM: combined quantum mechanics/molecular mechanics
RMSD: root mean square deviation

## References

[pcbi-0030021-b001] Alberts B, Bray D, Lewis J, Raff M, Roberts K (1994). Molecular biology of the cell. 3rd edition.

[pcbi-0030021-b002] Vale RD, Milligan RA (2000). The way things move: Looking under the hood of molecular motor proteins. Science.

[pcbi-0030021-b003] Bustamante C, Keller D, Oster G (2001). The physics of molecular motors. Acc Chem Res.

[pcbi-0030021-b004] Schliwa M (2002). Molecular motors.

[pcbi-0030021-b005] Volkman BF, Lipson D, Wemmer DE, Kern D (2001). Two-state allosteric behavior in a single-domain signaling protein. Science.

[pcbi-0030021-b006] Veenhoff LM, Heuberger EHML, Poolman B (2002). Quaternary structure and function of transport proteins. Trends Biochem Sci.

[pcbi-0030021-b007] Howard J (2001). Mechanics of motor proteins and the cytoskeleton.

[pcbi-0030021-b008] Gao Y, Karplus M (2004). Biomolecular motors: The F_1_-ATPase paradigm. Curr Opin Struct Biol.

[pcbi-0030021-b009] Wang HY, Oster G (1998). Energy transduction in the F_1_ motor of ATP synthase. Nature.

[pcbi-0030021-b010] Rees DC, Howard JB (1999). Structural bioenergetics and energy transduction mechanisms. J Mol Biol.

[pcbi-0030021-b011] Keike MC, Titus MA, Schliwa M (2002). The myosin superfamily: An overview. Molecular motors.

[pcbi-0030021-b012] Shih WM, Gryczynski Z, Lakowicz JR, Spudich JA (2000). A FRET-based sensor reveals large ATP hydrolysis-induced conformational changes and three distinct states of the molecular motor myosin. Cell.

[pcbi-0030021-b013] Lymn RW, Taylor EW (1971). Mechanism of adenosine triphosphate hydrolysis by actomyosin. Biochem.

[pcbi-0030021-b014] Trentham DR, Eccleston JF, Bagshaw CR (1976). Kinetic-analysis of ATPase mechanisms. Q Rev Biophys.

[pcbi-0030021-b015] Geeves MA, Holmes KC (1999). Structural mechanism of muscle contraction. Annu Rev Biochem.

[pcbi-0030021-b016] Holmes KC (2004). Introduction. Proceedings of a discussion meeting held at the Royal Society on 17 and 18 May 2004. Phil Trans R Soc Lond B Biol Sci.

[pcbi-0030021-b017] Geeves MA, Holmes KC (2005). The molecular mechanism of muscle contraction. Adv Prot Chem.

[pcbi-0030021-b018] Bauer CB, Holden HM, Thoden JB, Smith R, Rayment I (2000). X-ray structures of the Apo and MgATP-bound states of Dictyostelium discoideum myosin motor domain. J Biol Chem.

[pcbi-0030021-b019] Smith CA, Rayment I (1996). X-ray structure of the Magnesium(II)·ADP·Vanadate complex of the Dictyostelium discoideum myosin motor domain to 1.9 Å resolution. Biochemistry.

[pcbi-0030021-b020] Rayment I (1996). The structural basis of the myosin ATPase activity. J Biol Chem.

[pcbi-0030021-b021] Onishi H, Kojima S, Katoh K, Fujiwara K, Martinez HM (1998). Functional transitions in myosin: Formation of a critical salt-bridge and transmission of effect to the sensitive tryptophan. Proc Natl Acad Sci U S A.

[pcbi-0030021-b022] Onishi H, Ohki T, Mochizuki N, Morales MF (2002). Early stages of energy transduction by myosin: Roles of Arg in switch I, of Glu in switch II, and of the salt-bridge between them. Proc Natl Acad Sci U S A.

[pcbi-0030021-b023] Ito K, Uyeda QP, Suzuki Y, Sutoh K, Yamamoto K (2003). Requirement of domain–domain interaction for conformational change and functional ATP hydrolysis in myosin. J Biol Chem.

[pcbi-0030021-b024] Sasaki N, Ohkura R, Sutoh K (2003). Dictyostelium myosin II mutations that uncouples the converter swing and ATP hydrolysis cycle. Biochem.

[pcbi-0030021-b025] Highsmith S, Polosukhina K, Eden D (2000). Myosin motor domain lever arm rotation is coupled to ATP hydrolysis. Biochem.

[pcbi-0030021-b026] Málnási-Csizmadia A, Woolley RJ, Bagshaw CR (2000). Resolution of conformational states of *Dictyostelium* myosin II motor domain using tryptophan (W501) mutants: Implications for the open–closed transition identified by crystallography. Biochem.

[pcbi-0030021-b027] Málnási-Csizmadia A, Pearson DS, Kovács M, Woolley RJ, Geeves MA (2001). Kinetic resolution of a conformational transition and the ATP hydrolysis step using relaxation methods with a *Dictyostelium* myosin II mutant containing a single tryptophan residue. Biochemistry.

[pcbi-0030021-b028] Málnási-Csizmadia A, Kovács M, Woolley RJ, Botchway SW, Bagshaw CR (2001). The dynamics of the relay loop tryptophan residue in the Dictyostelium myosin motor domain and the origin of spectroscopic signals. J Biol Chem.

[pcbi-0030021-b029] Li GH, Cui Q (2004). Mechanochemical coupling in myosin: A theoretical analysis with molecular dynamics and combined QM/MM reaction path calculations. J Phys Chem B.

[pcbi-0030021-b030] Fischer S, Windshugel B, Horak D, Holmes KC, Smith JC (2005). Structural mechanism of the recovery stroke in the myosin molecular motor. Proc Natl Acad Sci U S A.

[pcbi-0030021-b031] Schwarzl SM, Smith JC, Fischer S (2006). Insights into the chemomechanical coupling of the myosin motor from simulation of its ATP hydrolysis mechanism. Biochem.

[pcbi-0030021-b032] Koppole S, Smith JC, Fischer S (2006). Simulations of the myosin II motor reveal a nucleotide-state sensing element that controls the recovery stroke. J Mol Biol.

[pcbi-0030021-b033] Eisenberg E, Hill TL (1985). Muscle contraction and free energy transduction in biological systems. Science.

[pcbi-0030021-b034] Torrie GM, Valleau JP (1977). Non-physical sampling distributions in Monte-Carlo free-energy estimation: Umbrella sampling. J Comput Phys.

[pcbi-0030021-b035] Allen MP, Tildesley DJ (1987). Computer simulation of liquids.

[pcbi-0030021-b036] Yu H, Ma L, Yang Y, Cui Q (2006). Mechanochemical coupling in the myosin motor domain. II. Analysis of critical residues. PLoS Comput Biol.

[pcbi-0030021-b037] Schlitter J, Engels M, Krüger P, Jacoby E, Wollmer A (1993). Targeted molecular dynamics simuation of conformational change: Application to the T ↔ R transition in insulin. Mol Simu.

[pcbi-0030021-b038] Schlitter J, Engels M, Krüger P (1994). Targeted molecular-dynamics: A new approach for searching pathways of conformational transitions. J Mol Graph.

[pcbi-0030021-b039] Case DA (1994). Normal-mode analysis of protein dynamics. Curr Opin Struct Biol.

[pcbi-0030021-b040] Cui Q, Bahar I (2005). Normal mode analysis: Theory and applications to biological and chemical systems. Mathematical and Computational Biology Series.

[pcbi-0030021-b041] Lockless SW, Ranganathan R (1999). Evolutionarily conserved pathways of energetic connectivity in protein families. Science.

[pcbi-0030021-b042] Suel GM, Lockless SW, Wall MA, Ranganathan R (2003). Evolutionarily conserved networks of residues mediate allosteric communication in proteins. Nat Struct Biol.

[pcbi-0030021-b043] Li GH, Cui Q (2004). Analysis of functional motions in brownian molecular machines with an efficient block normal mode approach: Myosin-II and Ca^2+^-ATPase. Biophys J.

[pcbi-0030021-b044] Cui Q, Li GH, Ma JP, Karplus M (2004). A normal mode analysis of structural plasticity in the biomolecular motor F_1_-ATPase. J Mol Biol.

[pcbi-0030021-b045] Gao YQ, Yang W, Karplus M (2005). A structure-based model for the synthesis and hydrolysis of ATP by F_1_-ATPase. Cell.

[pcbi-0030021-b046] Yu J, Ha T, Schulten K (2006). Structure-based model of the stepping motor of PcrA helicase. Biophys J.

[pcbi-0030021-b047] Coureux PD, Sweeney HL, Houdusse A (2004). Three myosin V structures delineate essential features of chemo–mechanical transduction. EMBO J.

[pcbi-0030021-b048] Conibear PB, Málnási-Csizmadia A, Bagshaw CR (2004). The effect of F-actin on the relay helix position of myosin II, as revealed by tryptophan fluorescence, and its implications for mechanochemical coupling. Biochem.

[pcbi-0030021-b049] Cui Q (2006). Theoretical analysis of mechanochemical coupling in myosin. Roy Soc Chem (Special issue).

[pcbi-0030021-b050] Ramachandran GN, Sasisekharan V (1968). Conformation of polypeptides and proteins. Adv Prot Chem.

[pcbi-0030021-b051] Hovmoller S, Zhou T, Ohlson T (2002). Conformations of amino acids in proteins. Acta Cryst D.

[pcbi-0030021-b052] Shimada T, Sasaki N, Ohkura R, Sutoh K (1997). Alanine scanning mutagenesis of the switch I region in the ATPase site of Dictyostelium discoideum myosin II. Biochem.

[pcbi-0030021-b053] MacKerell AD, Bashford D, Bellott M, Dunbrack RL, Evanseck JD (1998). All-atom empirical potential for molecular modeling and dynamics studies of proteins. J Phys Chem B.

[pcbi-0030021-b054] Hill TL (1977). Free energy transduction in biology.

[pcbi-0030021-b055] Hill TL, Eisenberg E (1981). Can free-energy transduction be localized at some crucial part of the enzymatic cycle. Q Rev Biophys.

[pcbi-0030021-b056] Boyer PD (1993). The binding change mechanism for ATP synthase—Some probabilities and possibilities. Biochim Biophys Acta.

[pcbi-0030021-b057] Cui Q (2006). Theoretical and computational studies of vectorial processes in biomolecules. Theo Chem Acc.

[pcbi-0030021-b058] Fox RF (1998). Rectified brownian movement in molecular and cell biology. Phys Rev E.

[pcbi-0030021-b059] Navizet I, Lavery R, Jernigan RL (2004). Myosin flexibility: Structural domains and collective vibrations. Prot Struct Func Bioinform.

[pcbi-0030021-b060] Bahar I, Rader AJ (2005). Coarse-grained normal mode analysis in structural biology. Curr Opin Struct Biol.

[pcbi-0030021-b061] Ma JP (2005). Usefulness and limitations of normal mode analysis in modeling dynamics of biomolecular complexes. Structure.

[pcbi-0030021-b062] Tama F, C L Brooks III (2006). Symmetry, form, and shape: Guiding principles for robustness in macromolecular machines. Annu Rev Biophys Biomol Struct.

[pcbi-0030021-b063] Zheng WJ, Brooks BR, Thirumalai D (2006). Low-frequency normal modes that describe allosteric transitions in biological nanomachines are robust to sequence variations. Proc Natl Acad Sci U S A.

[pcbi-0030021-b064] Purcell TJ, Sweeney HL, Spudich JA (2005). A force-dependent state controls the coordination of processive myosin V. Proc Natl Acad Sci U S A.

[pcbi-0030021-b065] Reubold TF, Eschenburg S, Becker A, Kull FJ, Manstein DJ (2003). A structural model for actin-induced nucleotide release in myosin. Nat Struct Biol.

[pcbi-0030021-b066] Coureux PD, Wells AL, Menetry J, Yengo CM, Morris CA (2003). A structural state of the myosin V motor without bound nucleotide. Nature.

[pcbi-0030021-b067] Lan GH, Sun SX (2005). Dynamics of myosin-V processivity. Biophys J.

[pcbi-0030021-b068] Lan GH, Sun SX (2005). Dynamics of myosin-driven skeletal muscle contraction: I. Steady-state force generation. Biophys J.

[pcbi-0030021-b069] Houdusse A, Sweeney HL (2001). Myosin motors: Missing structures and hidden springs. Curr Opin Struct Biol.

[pcbi-0030021-b070] Brooks BR, Bruccoleri RE, Olafson BD, States DJ, Swaminathan S (1983). CHARMM—A program for macromolecular energy, minimization, and dynamics calculations. J Comput Chem.

[pcbi-0030021-b071] Fiser A, Do RKG, Sali A (2000). Modeling of loops in protein structures. Prot Sci.

[pcbi-0030021-b072] Fiser A, Sali A (2003). ModLoop: Automated modeling of loops in protein structures. Bioinformatics.

[pcbi-0030021-b073] Jorgensen WL, Chandrasekhar J, Madura JD, Impey RW, Klein ML (1983). Comparison of simple potential functions for simulating liquid water. J Chem Phys.

[pcbi-0030021-b074] Brunger AT, Karplus M (1988). Polar hydrogen positions in proteins: Empirical energy placement and neutron-diffraction comparison. Proteins.

[pcbi-0030021-b075] Brooks CL, Karplus M (1989). Solvent effects on protein motion and protein effects on solvent motion: Dynamics of the active-site region of lysozyme. J Mol Biol.

[pcbi-0030021-b076] Ryckaert JP, Ciccotti G, Berendsen HJC (1977). Numerical integration of cartesian equations of motion of a system with constraints—Molecular dynamics of n-alkanes. J Comput Phys.

[pcbi-0030021-b077] Steinbach PJ, Brooks BR (1994). New spherical-cutoff methods for long-range forces in macromolecular simulation. J Comput Chem.

[pcbi-0030021-b078] Beglov D, Roux B (1994). Finite representation of an infinite bulk system: Solvent boundary potential for computer-simulations. J Chem Phys.

[pcbi-0030021-b079] Im W, Berneche S, Roux B (2001). Generalized solvent boundary potential for computer simulations. J Chem Phys.

[pcbi-0030021-b080] Nina M, Beglov D, Roux B (1997). Atomic radii for continuum electrostatics calculations based on molecular dynamics free energy simulations. J Phys Chem B.

[pcbi-0030021-b081] Nina M, Im W, Roux B (1999). Optimized atomic radii for protein continuum electrostatics solvation forces. Biophys Chem.

[pcbi-0030021-b082] Roux B (1995). The calculation of the potential of mean force using computer-simulations. Comput Phys Commun.

[pcbi-0030021-b083] Banavali NK, Roux B (2005). Free energy landscape of A-DNA to B-DNA conversion in aqueous solution. J Am Chem Soc.

[pcbi-0030021-b084] Banavali NK, Roux B (2005). The N-terminal end of the catalytic domain of Src kinase Hck is a conformational switch implicated in long-range allosteric regulation. Structure.

[pcbi-0030021-b085] Kumar S, Bouzida D, Swendsen RH, Kollman PA, Rosenberg JM (1992). The weighted histogram analysis method for free-energy calculations on biomolecules. I. The method. J Comput Chem.

[pcbi-0030021-b086] Kumar S, Rosenberg JM, Bouzida D, Swendsen RH, Kollman PA (1995). Multidimensional free-energy calculations using the weighted histogram analysis method. J Comput Chem.

[pcbi-0030021-b087] Warshel A, Levitt M (1976). Theoretical studies of enzymic reactions—Dielectric, electrostatic and steric stabilization of carbonium-ion in reaction of lysozyme. J Mol Biol.

[pcbi-0030021-b088] Field MJ, Bash PA, Karplus M (1990). A combined quantum-mechanical and molecular mechanical potential for molecular-dynamics simulations. J Comput Chem.

[pcbi-0030021-b089] Elstner M, Porezag D, Jungnickel G, Elsner J, Haugk M (1998). Self-Consistent-Charge Density-Functional Tight-Binding method for simulations of complex materials properties. Phys Rev B.

[pcbi-0030021-b090] Cui Q, Elstner M, Kaxiras E, Frauenheim T, Karplus M (2001). A QM/MM implementation of the self-consistent charge density functional tight binding (SCC-DFTB) method. J Phys Chem B.

[pcbi-0030021-b091] Reuter N, Dejaegere A, Maigret B, Karplus M (2000). Frontier bonds in QM/MM methods: A comparison of different approaches. J Phys Chem A.

[pcbi-0030021-b092] Das D, Eurenius KP, Billings EM, Sherwood P, Chatfield DC (2002). Optimization of quantum mechanical molecular mechanical partitioning schemes: Gaussian delocalization of molecular mechanical charges and the double link atom method. J Chem Phys.

[pcbi-0030021-b093] König PH, Hoffmann M, Frauenheim T, Cui Q (2005). A critical evaluation of different QM/MM frontier treatments with SCC-DFTB as the QM method. J Phys Chem B.

[pcbi-0030021-b094] Schaefer P, Riccardi D, Cui Q (2005). Reliable treatment of electrostatics in combined QM/MM simulation of macromolecules. J Chem Phys.

