# Supplementary material for: Mechanochemical Coupling in the Myosin Motor Domain. I. Insights from Equilibrium Active-Site Simulations
Source: PLoS Comput Biol. 2007 Feb 9;3(2):e21. doi: 10.1371/journal.pcbi.0030021 (PMC1796662; doi:10.1371/journal.pcbi.0030021)
Supplement: Supplementary file 1 [file pcbi.0030021.sd001.pdf]

Protocol S1: Mechanochemical coupling in the myosin motor domain. I. Insights from equilibrium active-site simulations

A Sidechain distribution of P-loop, Switch I and Switch II

To investigate the effect of ATP hydrolysis on the local flexibility of active-site residues, the sidechain distributions (Fig. S1-S3) are analyzed for both the ATP and ADP·P_i state with the 1VOM simulations (closed active-site). In addition, the same quantities are also analyzed for the 1VOM-ATP simulation with a larger inner region (32 Å instead of 20 Å) to investigate whether the size of the GSBP inner region has a major impact on the active-site flexibility. As seen in Fig. S1-S3, little difference is found between simulations of different inner region size, except for a few residues and the effect is small in all cases. As to the effect of ATP hydrolysis, the only residue that has been substantially affected is Ser 237, which has a much broader distribution in the ADP·P_i state, suggesting that the interaction with Mg^{2+} is substantially reduced.

B Convergence of the active-site open/close PMFs

As shown in Fig. S4, the calculated PMFs with nearly 4-5 times more simulation data are essentially identical with the original PMFs. This ensures that the computed PMFs are converged to a satisfactory degree.

C Figure Captions

Fig.S1-S3 Sidechain distributions in the P-loop (Fig. S1), Switch I (Fig. S2) and Switch II (Fig. S3) residues from different simulations.

Fig. S4 Potentials of mean force for the open/close of the active site in the (a) 1FMW-ATP and (b) 1VOM-ATP simulations with different length of trajectories.

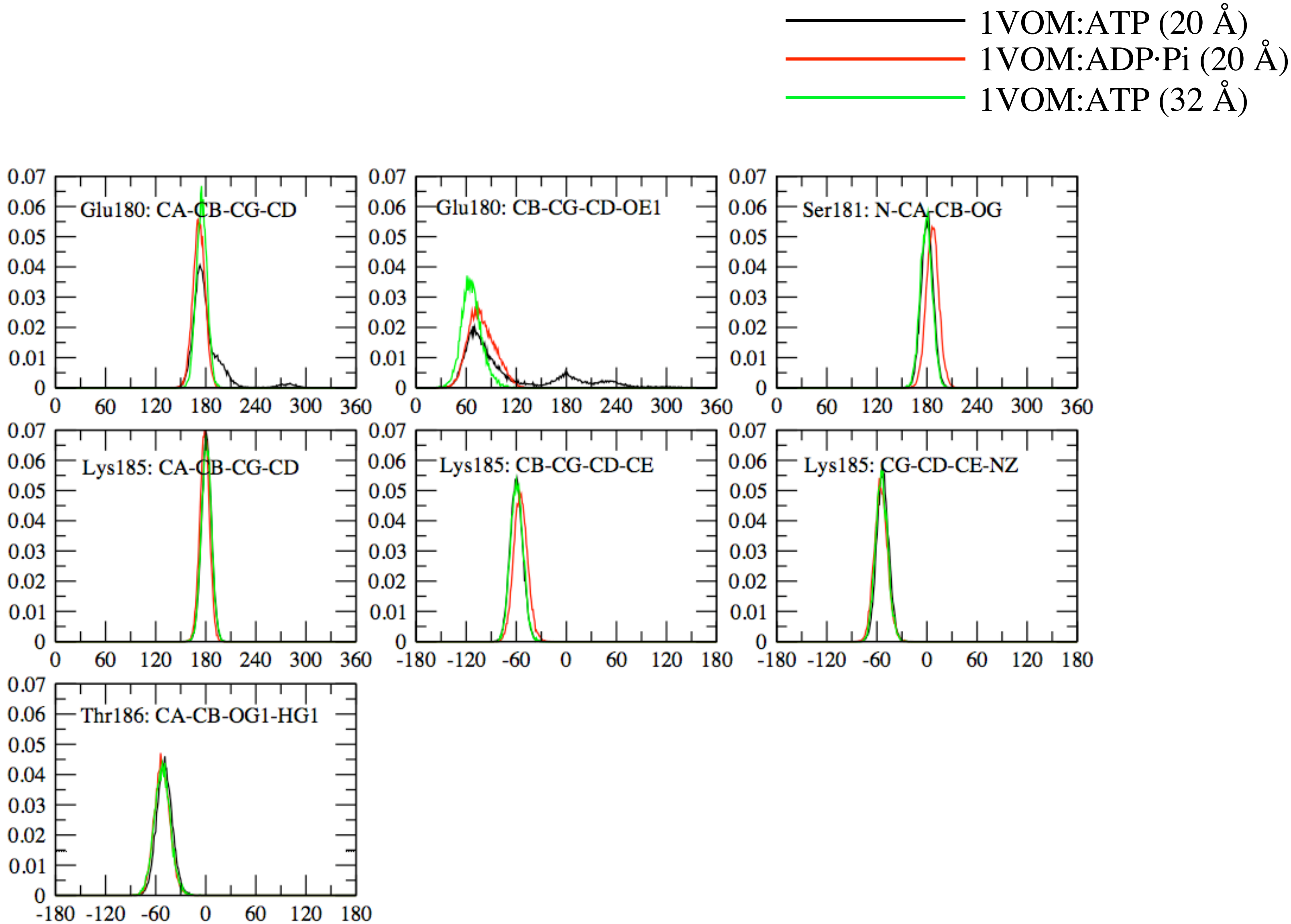
1VOM:ATP (20 Å)
1VOM:ADP·Pi (20 Å)
1VOM:ATP (32 Å)
Glu180: CA-CB-CG-CD
Glu180: CB-CG-CD-OE1
Ser181: N-CA-CB-OG
Lys185: CA-CB-CG-CD
Lys185: CB-CG-CD-CE
Lys185: CG-CD-CE-NZ
Thr186: CA-CB-OG1-HG1

Fig. S1

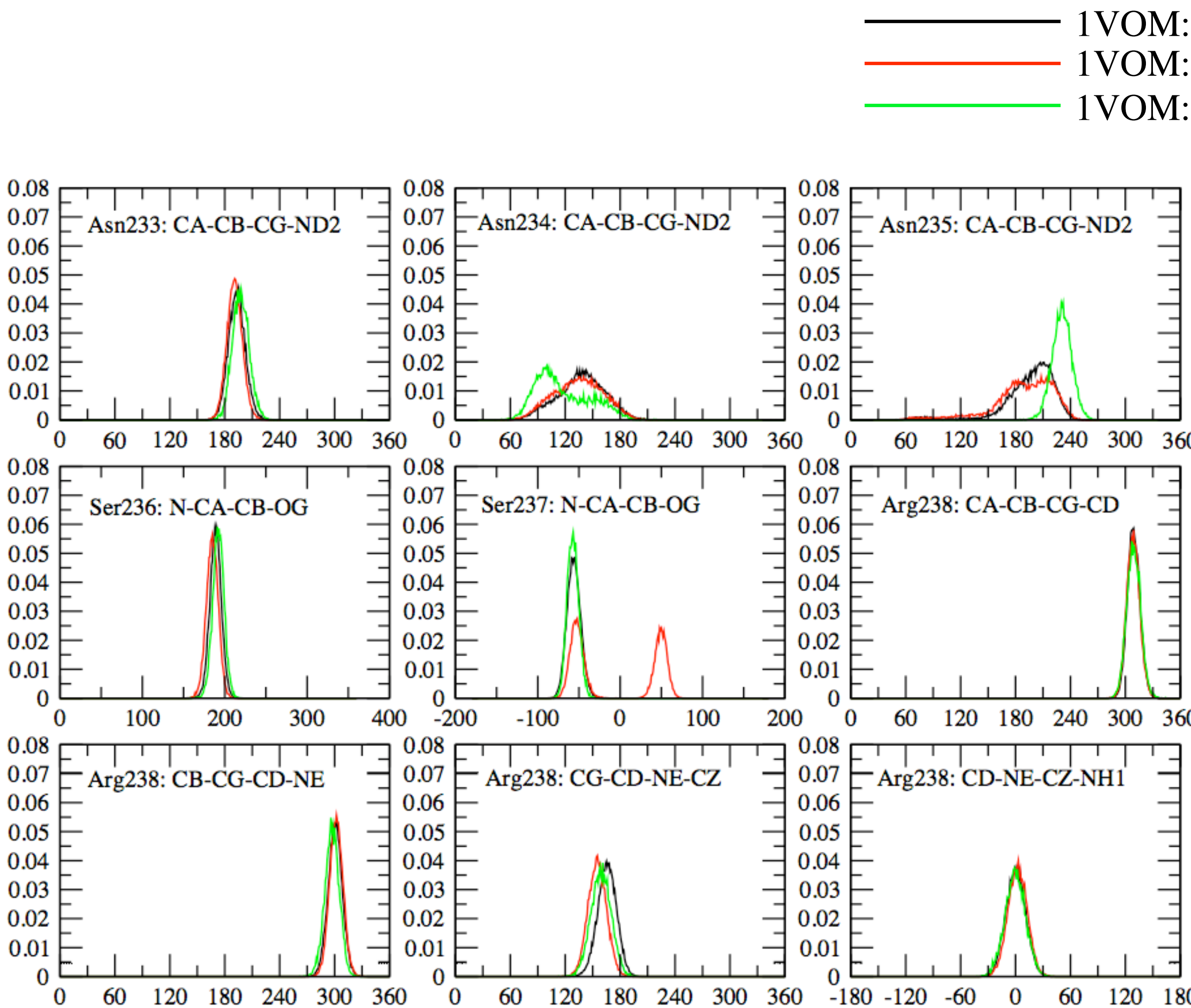
1VOM:ATP (20 Å)
1VOM:ADP·Pi (20 Å)
1VOM:ATP (32 Å)
Asn233: CA-CB-CG-ND2
Asn234: CA-CB-CG-ND2
Asn235: CA-CB-CG-ND2
Ser236: N-CA-CB-OG
Ser237: N-CA-CB-OG
Arg238: CA-CB-CG-CD
Arg238: CB-CG-CD-NE
Arg238: CG-CD-NE-CZ
Arg238: CD-NE-CZ-NH1

Fig. S2

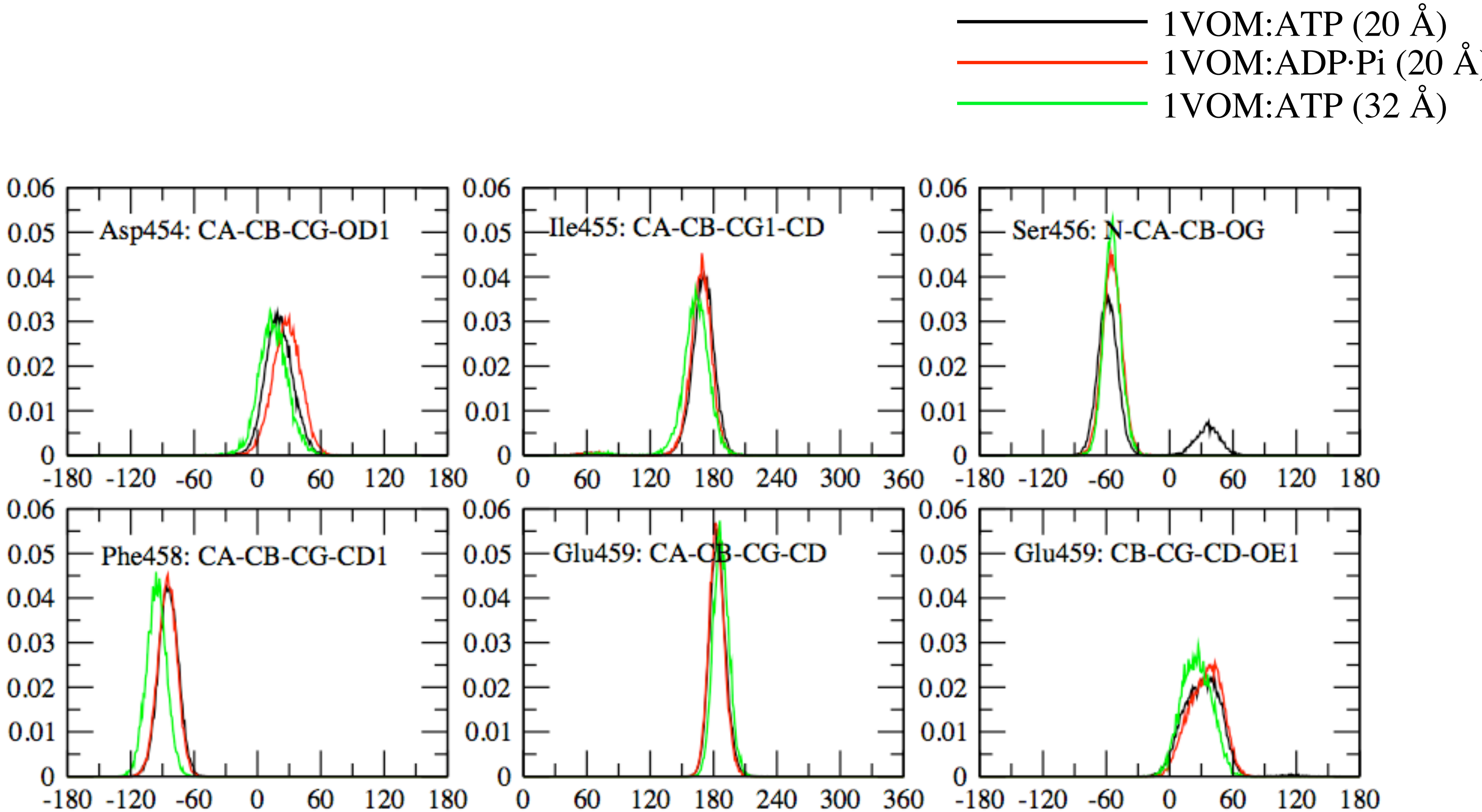

1VOM:ATP (20 Å)
1VOM:ADP·Pi (20 Å)
1VOM:ATP (32 Å)
Asp454: CA-CB-CG-OD1
Ile455: CA-CB-CG1-CD
Ser456: N-CA-CB-OG
Phe458: CA-CB-CG-CD1
Glu459: CA-CB-CG-CD
Glu459: CB-CG-CD-OE1

Fig. S3

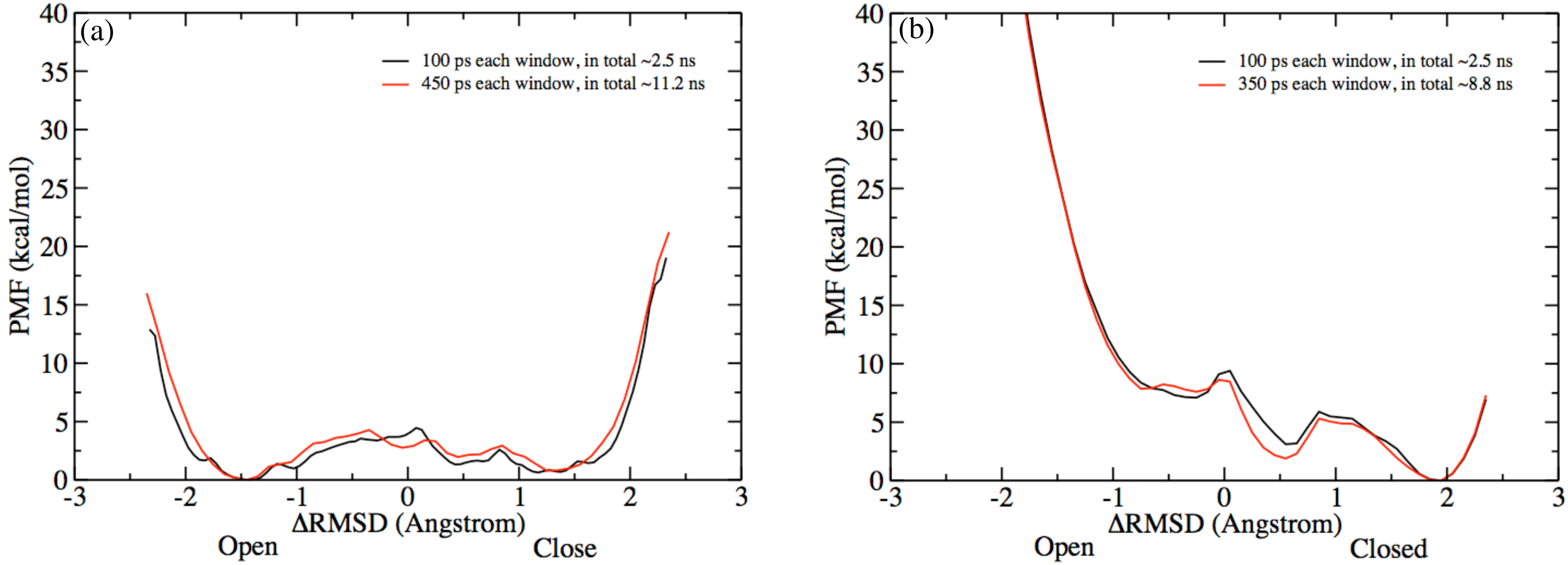
(a)
100 ps each window, in total ~2.5 ns
450 ps each window, in total ~11.2 ns
PMF (kcal/mol)
ΔRMSD (Angstrom)
Open
Close
(b)
100 ps each window, in total ~2.5 ns
350 ps each window, in total ~8.8 ns
PMF (kcal/mol)
ΔRMSD (Angstrom)
Open
Closed

Fig. S4
